# Effects of Cassava and Modified Starch on the Structural and Functional Characteristics of Peanut Protein-Based Meat Analogs

**DOI:** 10.3390/foods14162849

**Published:** 2025-08-17

**Authors:** Yuhan Su, Jiale Guan, Shuhong Liu, Yiqun Zhu, Liangyan Hu, Yifan Zhang, Fei Lu, Minpeng Zhu

**Affiliations:** College of Grain Science and Technology, Shenyang Normal University, Shenyang 110034, China; yuhans226@163.com (Y.S.); gl86007639@163.com (J.G.); liushuhong1230@163.com (S.L.); zyq06101917@163.com (Y.Z.); 13840202809@163.com (L.H.); even.home@163.com (Y.Z.)

**Keywords:** plant-based meat analog, high-moisture extrusion technology, protein–starch interaction, cassava starch, functional properties

## Abstract

Meat analog manufacturing via high-moisture extrusion technology is a complex process wherein the properties of protein materials constitute a critical determining factor. In this study, we enhanced the fiber structure properties of high-moisture extruded peanut protein-based meat analogs by incorporating different starches (cassava starch, acetyl distarch phosphate [ADSP], and hydroxypropyl starch) to address challenges in water retention, emulsification, and digestibility. The impact of the starch content (0, 3, 6, 9, 12%) was assessed using low-field nuclear magnetic resonance, ultraviolet/fluorescence spectroscopy, differential scanning calorimetry, sodium dodecyl sulfate–polyacrylamide gel electrophoresis, and functional tests. Compared with controls without starch, adding 6% ADSP significantly improved the water retention by forming a dense, charged network, reducing T_2b_ (0.37 ms) and T_22_ (175.30 ms). ADSP (12%) enhanced the emulsification (activity index 10.28 m^2^/g, stability index 75%); the cassava starch (12%) increased the in vitro protein digestibility to 83% due to amylopectin degradation. Hydroxypropyl starch (6%) elevated the thermal stability (peak temperature 125.71 °C) by forming a viscous protective matrix (*p* < 0.05). Ultraviolet and fluorescence spectra indicated protein–starch interactions, with ADSP inducing the most pronounced conformational changes. This study demonstrated that the starch type and concentration critically modulate protein–starch interactions, offering guidance for enhancing the quality of meat analogs.

## 1. Introduction

The United Nations Department of Economic and Social Affairs published a report in November 2024 stating that the global population is expected to reach 10.8 billion by 2080 [1]. With the food demand growing from 55% to 65%, the meat demand will reach 560–620 million tons [2], but the livestock industry is expected to encounter considerable challenges in meeting this demand. Increasing the production of meat analogs, comprising plant-based proteins, may offer a practical solution to help meet the demand. The global market for plant-based meat analogs is projected to reach USD 10 billion by 2025 and USD 10.35 billion by 2031. Surveys have indicated that compared to consumers in the United States (74.7%), those in China (95.6%) and India (94.5%) have a higher acceptance rate for plant-based meat analogs [3]. Consequently, Asian countries, notably China, are poised to offer substantial market prospects for meat analogs.

Despite the potential benefits of meat analogs, plant proteins alone are incapable of fully replicating meat characteristics [4]. The structural characteristics of muscle fibers, namely their arrangement and water retention capacity, are widely regarded as defining meat characteristics [5]. However, extruded meat analogs do not exhibit the three-dimensional anisotropic structure of muscle fibers or bundles. Furthermore, enhancing the sensory characteristics of meat analogs, particularly their juiciness and texture, continues to represent a substantial challenge in this field.

High-moisture extrusion (HME) is a promising technology for developing meat analogs because of its high production efficiency, nutritional component retention, low energy consumption, lack of exhaust emissions, and ability to produce a fibrous structure similar to that of animal meat [6,7]. HME involves the application of a high temperature, shear force, and pressure. These conditions result in protein denaturation, dissociation, recombination, and cross-linking, forming a protein matrix with anisotropic fiber structures [8]. Furthermore, when the temperature is elevated and the heating time reduced, the energy utilization is more efficient, while antinutritional factors are reduced and nutrients are preserved. Despite the promising characteristics of HME, further improvements are needed in terms of process optimization, the nutritional and functional balance, energy efficiency and sustainability, and raw material adaptability. In particular, considering the limited fibrotic effect of HME on certain plant proteins, pre-treatment methods or additive technologies are needed to broaden the applicability of raw materials.

Researchers have incorporated various edible polymers to enhance product quality [9]. Starch, a polysaccharide that is both economically and functionally diverse, possesses several relevant characteristics, including the ability to thicken, gel, adhere, cause dandruff, swell, and undergo gelatinization. Starch has a multifaceted effect on the water-holding capacity and texture. In addition, starch affects the emulsification, thermal stability, and digestive properties of products [10]. Vegetable proteins and starch agglutinate into particles and anisotropic fibrils through physicochemical interactions, thereby endowing meat with the desired texture and mouthfeel.

In the standard extrusion process for manufacturing meat analogs, proteins are frequently processed in conjunction with starch under elevated temperatures and high levels of shear force to yield a composite fibrous meat-like texture [11]. The molecular degradation and gelatinization of starch during the squeezing process may promote protein digestion, as the degradation products (dextrin and reducing sugars) serve as substrates for enzymes, enhancing protein digestibility [12]. The extrudates of starch-containing mixtures undergo pasting during extrusion, increasing the degree of plastic deformation. This, in turn, promotes protein encapsulation in the starch matrix and has a corresponding effect on protein denaturation and digestibility [13]. Starch pasting promotes protein denaturation, resulting in structural changes, which is attributed to the ability of elevated temperatures and mechanical shear to disrupt hydrogen bonding and protein hydrophobicity. Denaturation enhances protein digestibility; however, it concomitantly results in protein aggregation and precipitation, reducing solubility [14]. Guo et al. [15] reported that the thermal properties of pea starch allow it to readily polymerize heated peanut protein, altering the secondary protein structure. Dobson et al. [16] found that pea protein containing 70% thermally inhibited starch had a lower hydration ability, meaning that the protein readily formed a tight structure. Cassava starch (CS) exhibits a high number of hydrogen bonds within and between molecules, a high amylopectin content, a low gelatinization temperature, an early water absorption and swelling capacity, and the potential to enhance the water and oil absorption of meat analogs. Acetyl distarch phosphate (ADSP) has advantageous gel properties and is used as a gel, water retention agent, thickener, and excipient in meat analogs [17]. Hydroxypropyl starch (HS) is highly viscous and stable at elevated temperatures. This process occurs in conjunction with thermal protein denaturation, enhancing the stability of the protein network [18]. All three types of starches have the potential to enhance the quality of meat analogs.

Plant-based meat analogs produced using a single plant protein have notable shortcomings in several key characteristics, including the color, flavor, and texture. Therefore, meat analogs tend to be produced using a mixture of different proteins. Soy protein isolate is commonly used as a raw material for producing meat analogs because it forms a fibrous structure. However, meat analogs containing a high proportion of soy protein isolate have a strong beany flavor and lower structural integrity [19]. Globally, >5 million tons of defatted peanut meal (with a protein content > 55%) is produced annually, most of which is used as animal feed, leading to resource underutilization. Peanut protein powder mainly consists of arachin and conarachin, has a light color, exhibits high digestibility (~90%), and lacks flatulence factors and the beany flavor that is prevalent with soy beans [20]. However, peanut protein exhibits a weaker gel-forming ability than soy protein isolate; thus, the texture of peanut protein after HME processing requires further improvement [21]. Consequently, further research on other plant proteins and complex food systems containing other polymers is needed. This study examined the effects of three starches and modified starches on the structural and functional characteristics of high-moisture extruded peanut tissue proteins. In addition, this study further substantiated the underlying causes and mechanisms of these effects to develop plant-based meat analogs with an enhanced quality.

## 2. Materials and Methods

### 2.1. Materials

Peanut protein powder (protein content 59.6%, dry basis) was obtained from Anyang Tianxiangrui Food Technology Co., Ltd. (Anyang, China); soybean protein isolate (protein content 87.4%, dry basis) was obtained from Shandong Yuwang Ecological Food Industrial Co., Ltd. (Yucheng, China); CS was obtained from Henan Wanbang Industrial Co., Ltd. (Zhengzhou, China); acetylated starch phosphate was obtained from Henan Wanbang Industrial Co., Ltd.; and HS was obtained from Youbaojia Food Co., Ltd. (Shangqiu, China). O-phthalaldehyde, pepsin, trypsin, and 2-mercaptoethanol (biological grade) were obtained from Shanghai Macklin Biochemical Co., Ltd. (Shanghai, China).

### 2.2. High-Moisture Extrusion

Extrusion trials were performed using a laboratory-scale co-rotating twin-screw extruder (UVGC-UVTE36, Hunan Chuangxiang Intelligent Technology Co., Ltd., Changsha, China) with a cooling die attached to the end of the twin screw. The screw diameter was 36 mm with a length-to-diameter ratio of 40:1. The extrusion barrel was divided into eight zones from the feed to the die. Each zone was temperature-controlled, heated separately using an electric cartridge system, and cooled with water. Finally, the extrusion barrel was equipped with an attached die with dimensions of 50 × 10 mm. According to our previous study, the process parameters of peanut protein/soy isolate protein extrusion experiments included the following: a peanut protein to soy isolate protein ratio of 4:1 (*w*/*w*), feed rate of 8.5 kg/h, feed moisture content of 60%, screw speed of 280 rpm, and mold cooling temperature of 55 °C. The temperatures of the material cylinder from the feeding area to the mold head area were 60, 80, 120, and 160 °C. To investigate the influence of starch, CS, ADSP, and HS were blended with peanut protein/soy isolate protein according to gradients of 0%, 3%, 6%, 9%, and 12% prior to the twin-screw extrusion experiments. After extrusion, all meat analogs were immediately sealed in zip-lock plastic bags and frozen at −20 °C for subsequent analysis.

### 2.3. Low-Field Nuclear Magnetic Resonance

A low-field nuclear magnetic resonance (LF-NMR) instrument (MesoMR23-060H-I-NMR, Jiangsu Newmai Co., Ltd., Suzhou, China) was used to ascertain the distribution of water within the samples, using a previously described method [22]. The freshly extruded meat analog samples were cut into 30 mm strips, wrapped in polytetrafluoroethylene film, and placed in a glass tube. The T_2_ relaxation times of the samples (T_2b_, T_21_, and T_22_) were measured using the CPMG pulse sequence. The following instrument operating parameters were used: wait time, 2000 ms; echo time, 0.15 ms; echo number, 2000 ms; and scan number, 8.

### 2.4. Ultraviolet Spectra

Ultraviolet (UV) spectra of the meat analogs were obtained using a Cary 5000 UV-Vis spectrophotometer (Agilent Technologies, Santa Clara, CA, USA) according to the procedure described by Agyare et al. [23]. The freeze-dried samples (0.25 g) were added to 20 mL of sodium phosphate buffer solution (0.04 mol/L, pH 7.5) and magnetically stirred for 1 h. The mixture was then centrifuged at 12,000 rpm for 15 min, and the supernatant was collected. The protein concentration in the supernatant was diluted five times before measurement. The supernatants were subjected to UV scanning at 200–400 nm.

### 2.5. Intrinsic Fluorescence Spectra

The tertiary structure of the proteins was analyzed using a fluorescence spectrophotometer (F97XP; Shanghai Ling Guang Technology Co., Ltd., Wuxi, China) [24]. The freeze-dried samples (0.25 g) were added to 20 mL of sodium phosphate buffer solution (0.04 mol/L) and magnetically stirred for 1 h. The mixture was then centrifuged at 12,000 rpm for 15 min, and the supernatant was collected. The protein concentration in the supernatant was diluted five times before measurement. The fluorescence spectrum was recorded at an excitation wavelength of 280 nm and an emission wavelength range of 300–500 nm.

### 2.6. Thermal Property Determination

According to Hossain et al. [25], the thermal properties of the samples were recorded using a differential scanning calorimeter (TA Instruments, New Castle, DE, USA). Freeze-dried samples (5.0 mg) were weighed in an aluminum pan, which was heated from 20 °C to 200 °C at a rate of 10 °C/min while being sealed. The nitrogen flow rate was maintained at 80 mL/min. The thermal transition temperature at its peak (Tp) and corresponding enthalpy change (ΔH) was measured.

### 2.7. Sodium Dodecyl Sulfate–Polyacrylamide Gel Electrophoresis

Protein subunits were analyzed through reducing and non-reducing sodium dodecyl sulfate–polyacrylamide gel electrophoresis (SDS-PAGE) [26], with some modifications. The gel used consisted of 12% separating and 5% stacking gels. Freeze-dried samples (6 mg) were mixed with 1 mL of deionized water. Five microliters of a mixture containing non-reducing loading buffer and mixture containing reducing loading buffer (after heating at 100 °C for 5 min) was loaded separately into the gel. The gel was stained with Coomassie blue fast staining solution for 4 min and de-stained with distilled water.

### 2.8. Thawing Loss

The meat analog sample (*W*_1_) was frozen at −20 °C for 7 d then thawed at 4 °C under sealed conditions for 8 h. After thawing completely, water was removed from the surface of the meat analogs with filter paper and weighed again (*W*_2_). Each sample was weighed in parallel five times. Thawing loss was calculated as follows:

(1) $$\mathrm{Thawing}\;\mathrm{loss}\;(\%)\;=\;\frac{W_{1}-W_{2}}{W_{1}}\;\times \;100$$

### 2.9. Emulsifying Properties

Emulsifying properties were determined according to the method described by Li et al. [27], with slight modifications. The meat analog samples were freeze-dried, ground, and sieved (80-mesh). The powder was dissolved in deionized water, and the resulting solution (15 mL, 1% w/v) was mixed with 5 mL of peanut oil, followed by homogenization at 10,000 r/m for 2 min. After standing for 0 and 10 min, 50 μL solution was taken from the bottom and added to 5 mL of 0.1% SDS solution. After swirling for 1 min, the absorbance was measured at 500 nm. Finally, the emulsifying activity index (EAI) and emulsifying stability index (ESI) were calculated using the following formulas.

(2) $$\mathrm{EAI}\;(\frac{m^{2}}{g})=\frac{2\;\times \;2.303\;\times \;N}{C\;\times \;\phi \;\times \;10^{4}}\;\times \;A_{0}$$

where C was the mass concentration of protein before emulsification, g/mL; ϕ was the volume fraction of oil in the sample, %; and N was the dilution factor 100.

(3) $$\mathrm{ESI}\;(\%)=\frac{A_{10}}{A_{0}}\;\times \;100$$

where *A*_10_ and *A*_0_ represent the absorbances of the 10 and 0 min emulsions, respectively.

### 2.10. In Vitro Protein Digestibility

The in vitro protein digestibility (IVPD) of both the physical mixture (peanut protein and soybean protein isolate, 4:1 *w*/*w*) and meat analog samples was determined using a pepsin–pancreatin system [28]. Briefly, 0.4 g sample was immersed in 80 mL of HCl containing 0.1 g pepsin, and the mixture was shaken for 2 h at 37 °C. Next, 1 mol/L NaOH was added to adjust the pH to 8.0 and inactivate the pepsin, whereafter pancreatin (0.5 g) was added to simulate small intestine digestion. After 2 h of incubation, the sample was treated with 5 mL of 10% trichloroacetic acid to stop the reaction, and the solution was centrifuged at 8000× *g* for 10 min. The IVPD was calculated based on the ratio of protein in the supernatant (*m*_1_) to the total protein in the sample (*m*_2_), according to the following equation:

(4) $$\mathrm{IVPD}\;(\%)\;=\;\frac{m_{1}}{m_{2}}\times 100$$

### 2.11. Statistical Analysis

All experiments were conducted in triplicate unless stated otherwise, and the results are presented as the mean ± standard deviation. Data analysis was conducted using Origin 2021 software (Origin Lab Inc., Northampton, MA, USA). Statistical significance was assessed using one-way analysis of variance in SPSS software (version 18.0; IBM, Armonk, NY, USA), with mean comparisons made using Duncan’s multiple range test (*p* < 0.05).

## 3. Results and Discussion

### 3.1. Water Distribution in Meat Analogs

**Figure 1:**
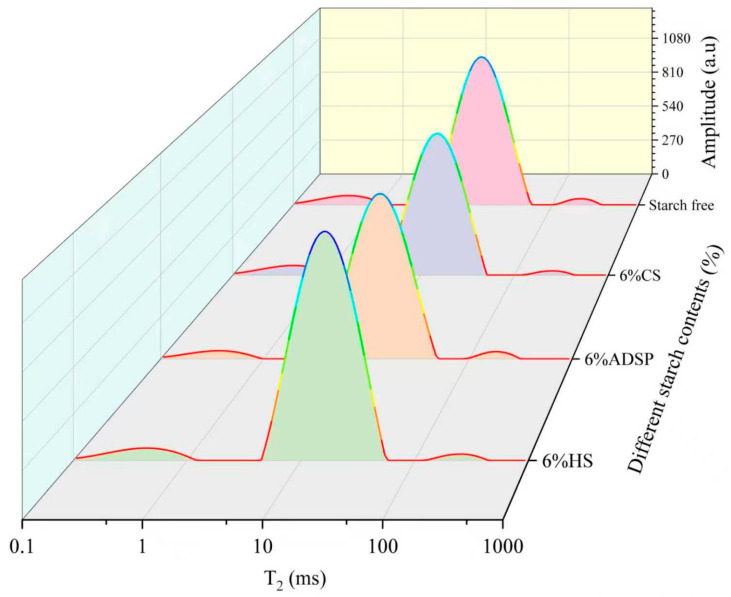
T_2_ relaxation time distribution curve of meat analogs with different starch contents. CS, cassava starch; ADSP, acetyl distarch phosphate; and HS, hydroxypropyl starch.

The T_2_ relaxation time distribution curves of meat analogs with varying starch contents, measured using LF-NMR, are presented in Figure 1 and Table 1.

ADSP exerted the most substantial influence on the water status and particularly reduced T_2b_, T_22_, T_21_, and M_21_, indicating that ADSP comprehensively restricted the water flow at all levels, thereby creating a highly constrained water network. The reduced T_22_ and increased M_21_ indicated a decrease in the degree of freedom of the free water in meat analogs, a reduction in the loss of bound water, and an improvement in the water retention capacity of meat analogs [29]. The capacity of both CS and HS to convert free water into other forms and act as water redistributors was evident from the significantly lower M_22_. The CS led to a significant increase in the M_2b_ content, whereas HS, similar to ADSP, significantly increased the content of M_21_ within the network. The significant reduction in the T_21_ and T_2b_ values of meat analogs indicates that it affected the water flow between protein macromolecules and improved the structural integrity of meat analogs. The density of plant-based meat analogs influences the moisture migration within their protein networks [30]. Tightly bound networks exhibit reduced moisture loss under extrusion conditions. The enhanced water-holding capacity was correlated with the decreased mobility of the aqueous phases within the matrix. The LF-NMR analysis revealed that samples with short T_2b_ relaxation times contained water protons in constrained molecular environments, reflecting a high structural density [31].

These findings may have been caused by ADSP being a chemically modified starch with negatively charged phosphate groups in its molecular chains. These hydrophilic groups act as powerful binding sites for water molecules. During extrusion, they form strong connections with polar groups or positively charged regions on protein chains through hydrogen bonds and electrostatic interactions. This cross-linking builds a denser and more robust protein–starch composite network than a pure protein network [32]. This mechanism effectively improved the water-holding capacity of the system, which is consistent with the observed reduction in the thawing loss. CS (rich in amylopectin) and HS (high viscosity at high temperatures) are primarily locked in water by forming a vast three-dimensional hydrogel network similar to a sponge [33]. During extrusion, these starch molecules undergo phase separation from the proteins, forming protein- and starch-rich phases. This sublayer transformation mechanism is key to forming fibrous structures and immobilizing water. This network effectively absorbs and fixes large amounts of free water and converts it into immobile water.

### 3.2. UV Spectral Analysis

**Figure 2:**
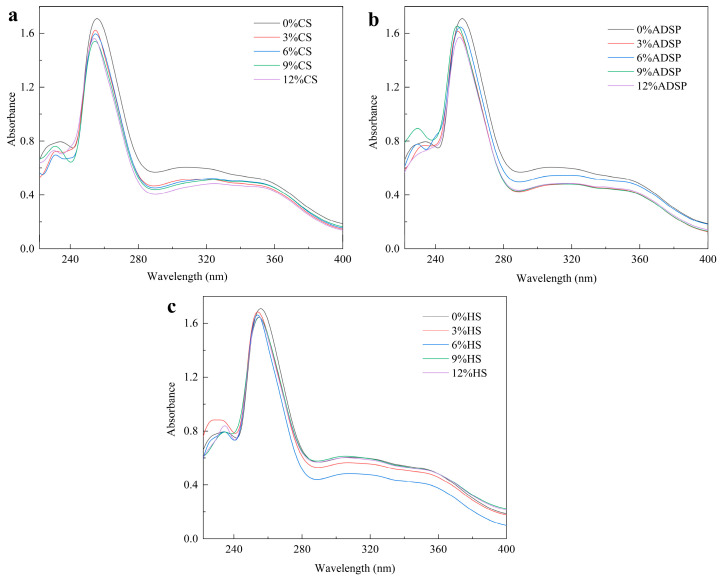
Ultraviolet spectra of meat analogs with different starch contents. (**a**) CS; (**b**) ADSP; and (**c**) HS.

The protein conformation was determined via UV spectrophotometry (Figure 2). All samples with added starch showed a lower UV absorption peak intensity at approximately 255 nm than that of the control group without starch. As the starch content increased, the absorption intensity decreased. FTIR data from our previous study confirmed the presence of absorption peaks at 1658, 1534, and 1440 cm^−1^, which were enhanced following the addition of the starch [34]. This not only improved the conformation of the protein, resulting in a blue shift in the maximum absorption wavelength (λ max; with the ADSP group showing the most significant blue shift), but also enhanced the interaction between the protein and starch through the Maillard reaction.

Tang et al. [35] showed that a decrease in the UV absorption intensity is typically attributed to a masking effect. This implies that protein molecules aggregate or form complexes with starch, causing some aromatic amino acid residues to be buried inside, thereby preventing them from absorbing UV light effectively. As the concentration of CS, ADSP, and HS increases, the peak absorbance in the spectrum exhibits a declining trend. This phenomenon can be attributed to the Maillard reaction occurring between proteins and low-molecular-weight reducing sugars generated during the gelatinization and breakdown of starch and the increase in the reducing sugar concentration reacting with increasing aromatic amino acids, resulting in a change in the protein spatial structure [36]. In comparison with meat analogs devoid of starch, the incorporation of starch leads to a maximum blue shift in the ultraviolet absorption wavelength, as starch promotes protein molecule depolymerization and expansion, generates new subunits, and increases the expansion degree of protein molecules, resulting in the blue shift in the maximum ultraviolet absorption wavelength [37]. The blue shift also indicated that the protein conformation changed from a planar state to a nonplanar state, and the steric effect could be attributed to aggregate formation.

### 3.3. Intrinsic Fluorescence Spectral Analysis

**Figure 3:**
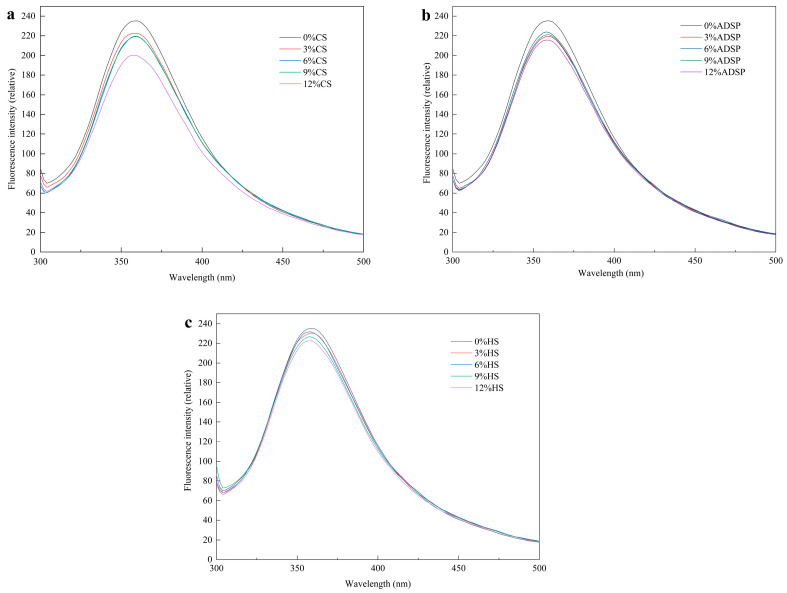
Fluorescence spectra of meat analogs with different starch contents. (**a**) CS; (**b**) ADSP; and (**c**) HS.

Hydrophobic interactions between the proteins were measured using fluorescence spectroscopy (Figure 3). The sample had a strong fluorescence peak at 360 nm, and compared with the content of no starch, the starch content reduced the fluorescence intensity of meat analogs, which was positively correlated with the content of CS and HS, and there was no significant change in the content of ADSP.

Li et al. [38] reported that a decrease in the fluorescence intensity (quenching) is direct evidence of stable protein–starch complex formation, which alters the energy transfer pathways of the fluorophores. Starch and tryptophan residues are hidden in the protein-formed polymer, enhancing the hydrophobic interaction between the starch and aromatic ring, resulting in a stronger fluorescence quenching of tyrosine and tryptophan and a decreased fluorescence intensity. Moreover, with an increase in the starch content, the maximum wavelength of meat analogs shows a slight blue shift. The maximum blue shift was 358 nm, a clear signal that the tryptophan residues, which contribute most to fluorescence, have moved from a relatively polar aqueous environment to a hydrophobic (nonpolar) microenvironment [39]. This may be because of polymerization, aggregation, or peptide–peptide associations between the starch and protein, resulting in a change in the tryptophan microenvironment, a decrease in the polarity of the fluorophore environment, the masking of some tryptophan residues in the protein in an environment with low polarity, and the shortening of the maximum wavelength [40]. The fluorescence spectra corresponded to the UV spectra. The starch content modulates protein aggregation during HME processing. Gelatinized starch chains intertwine with unfolded proteins, forming protein–starch complexes through steric hindrance and intermolecular interactions [41]. Starch-derived reducing sugars (e.g., dextrins and oligosaccharides) participate in the Maillard reaction with protein amino groups, yielding covalently linked glycosylated proteins [39]. This modification alters the polarity of amino acid residues and promotes the formation of high-molecular-weight polymers, corroborating the aggregation patterns observed in UV and fluorescence spectra [42].

### 3.4. Thermal Characteristic Analysis

Thermal transition properties of proteins were determined by DSC, as shown in Table 2. CS and ADSP had no significant effect on the Tp of the meat analogs, whereas HS had a significant effect on the Tp (*p* < 0.05). HS (6%) resulted in a dense meat analog structure, with compact proteins exhibiting a high Tp (125.71 °C). The ΔH represents the total energy required to disrupt the ordered structures of a protein (such as α-helices and β-sheets) and is, therefore, proportional to the degree of structural order and the strength of internal interactions [43]. The data show that the contents of HS had no significant effect on the ΔH. CS at a 9% content level significantly decreased the ΔH. Most notably, with a 3% content of ADSP, the ΔH jumped sharply and significantly from 28.69 J/g in the control group to 38.10 J/g, which is an increase of 33%. However, as the ADSP content increased to 9% and 12%, the ΔH value, though still higher than the control, showed a significantly decreasing trend. At 6%, HS significantly increased the thermal stability of the product. This indicates that at a specific concentration, HS effectively enhances the resistance of the protein network to high temperatures. At 3%, ADSP significantly increased the structural order of the protein matrix, forming a structure with stronger internal interactions. However, this promoting effect has an optimal concentration, as excessive ADSP (e.g., 12%) leads to a significant decrease in structural orderliness. Similarly, a high content of 9% CS also significantly reduced structural orderliness.

The changes in the Tp and ΔH are rooted in the differences in the composite network structure formed by the starch and protein during extrusion. The significant increase in the ΔH at a 3% ADSP content level strongly demonstrates that ADSP promotes the formation of ordered and stable protein–starch networks. The mechanism lies in the high reactivity of the phosphate groups on the ADSP molecular chains under a high temperature and pressure, which can act as cross-linkers to form a large number of new non-covalent bonds (such as hydrogen and ionic bonds) with polar or charged groups on the protein chains [44]. These additional interaction forces weave the protein molecules together in a tight and orderly manner, thus, requiring high energy (high ΔH) to disrupt and disintegrate this network. However, when the ADSP content is too high, the ΔH decreases. This reveals a supersaturation or dilution effect.

Excess starch molecules interact with each other and occupy space, interfering with and hindering the formation of an ideal ordered arrangement between protein molecules through steric hindrance [45]. At this point, the protein–protein interactions in the system are diluted by protein–starch interactions, leading to a decrease in the overall regularity of the network, thereby reducing the energy required to destroy it. This phenomenon is a typical manifestation of thermodynamic incompatibility in high-polymer physics at high concentrations, where the two polymers tend to phase separate rather than form a homogeneous mixed structure [41]. The HS significantly increased the thermal stability (Tp increased) without significantly changing the structural orderliness (ΔH remained largely unchanged). This reveals a stabilization mechanism that is completely different from that of ADSP. HS is known for its ability to maintain a high viscosity, even at high temperatures. During extrusion, HS may form a high-viscosity continuous phase that encapsulates and immobilizes protein aggregates. This viscous matrix acts as a kinetic barrier or thermal insulation layer, physically restricting the thermal motion and unfolding of protein segments [46]. Therefore, although the number and strength of chemical bonds within the protein (ΔH) did not change substantially, a high Tp is required to provide enough energy to overcome this kinetic barrier and cause the protein to denature.

### 3.5. SDS-PAGE Analysis

**Figure 4:**
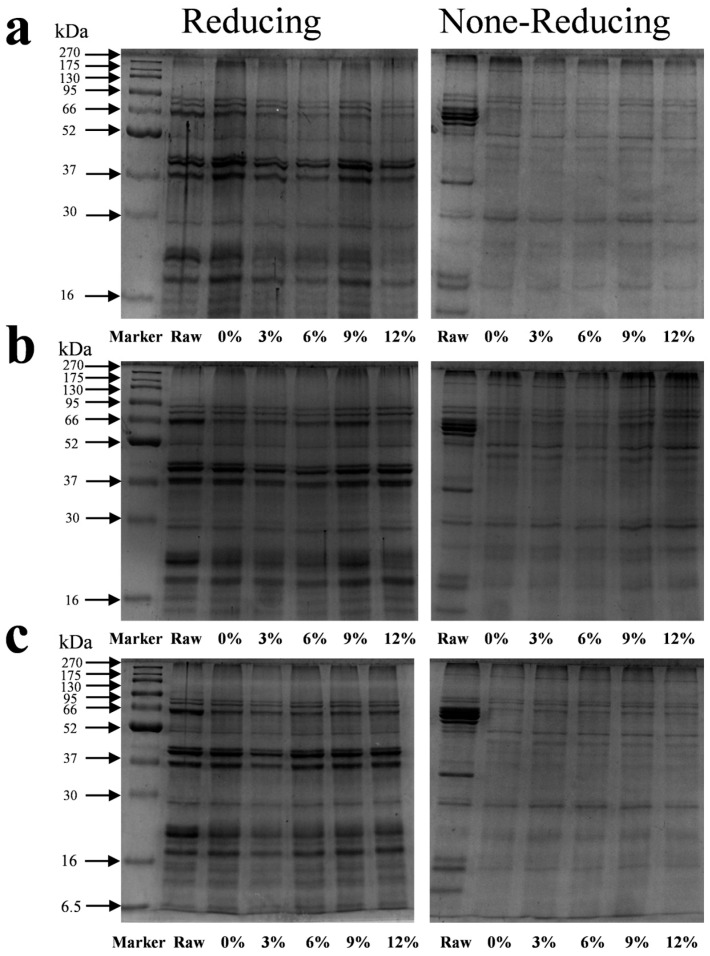
Sodium dodecyl sulfate polyacrylamide gel electrophoresis of different starch contents on meat analogs. (**a**) CS; (**b**) ADSP; and (**c**) HS.

The protein subunit composition and characteristics after protein denaturation were determined using SDS-PAGE (Figure 4). The marker bands in the first well were at 270, 175, 130, 95, 66, 52, 37, 30, 16, 15, and 6.5 kDa. The raw material and meat analog samples were mainly composed of 10 electrophoretic bands (subunits) with a molecular weight of 16–95 kDa. Compared to the untreated raw material, the protein subunit bands at approximately 37 and 66 kDa almost completely disappeared from the non-reducing gel after extrusion. All extruded samples showed significant protein retention in the loading wells and at the top of the stacking gel and could not enter the separating gel.

Under reducing conditions with β-mercaptoethanol, the 37 and 66 kDa bands, which were absent in the non-reduced state, reappeared but were of a significantly lower intensity compared to those of the raw material. All protein subunit bands were weaker than those of the raw material, and no new low-molecular-weight bands were detected. Overall, the content of the three types of starch had a minor effect on the electrophoretic profiles of the protein subunits. However, a subtle but consistent trend was that after adding 3% of CS, ADSP, and HS, the band density of the 23 kDa subunit was the lowest compared to the extruded sample without starch, with the effect of ADSP appearing particularly pronounced.

These changes in the molecular weight distribution reveal the core structural modifications of proteins during the extrusion process. The disappearance of bands under non-reducing conditions is the most direct evidence that proteins aggregate into large, insoluble aggregates through the formation of intermolecular disulfide bonds (–S–S–) [47]. These aggregates, owing to their excessively large molecular weights, cannot enter the pores of the polyacrylamide gel and, thus, appear to disappear from the electrophoretic gel. This confirms that the disulfide bond cross-linking is a key chemical reaction that imparts texture to the product during HME. Under reducing conditions, even though the disulfide bonds were broken, a large amount of protein could not enter the gel (as indicated by all band densities being lower than that of the raw material). This strongly suggested the formation of other types of stable covalent bonds in the presence of reducing agents. After increasing the starch content, the band density of the small 23 kDa subunit decreased, indicating that the starch content promoted the integration of these small subunits into the larger polymer network. The transformation of proteins from small soluble subunits to large insoluble polymers caused by the extrusion and starch content is a multistep, multifactor synergistic process. The high temperature (up to 160 °C) and strong shear force inside the extruder provide the energy for the protein denaturation. The native conformation of the protein is destroyed, and peptide chains unfold [48]. The unfolding of the peptide chains exposes the functional groups that were originally buried inside, especially the sulfhydryl groups (–SH) of the cysteine residues, to the reactive environment. The exposed sulfhydryl groups are easily oxidized at high temperatures, forming a disulfide bond (–S–S–) between the two sulfhydryl groups, thus covalently linking different protein chains to form a cross-linked network. This is the main reason for the disappearance of bands in non-reducing SDS-PAGE.

Increased thermal conditions can facilitate the dehydration–condensation reaction involving functional groups of amino acids, such as the interaction between lysine’s ε-amino moiety and carboxyl groups (γ or δ) of aspartate or glutamate. This leads to amide bond formation or Maillard reactions with sugars in the system to form stable covalent cross-links, which cannot be broken by reducing agents. Gelatinized starch molecular chains increase the viscosity and concentration of the molten material, creating a molecular crowding effect that forces protein molecules closer to each other, thereby increasing the probability of cross-linking reactions [49]. Simultaneously, the thermodynamic incompatibility between proteins and starch leads to phase separation, and the increased protein concentration at the phase interface facilitates aggregation and cross-linking [50]. The reducing sugars produced by starch degradation act as bridges for covalent cross-linking through the Maillard reaction, connecting one protein chain to another and thus linking the protein network more tightly. By altering the rheological properties of the melt and participating in chemical reactions, starch creates favorable conditions for the small-molecular-weight 23 kDa subunit to be incorporated into the growing polymer network. This can be achieved through enhanced hydrophobic interactions or direct chemical bonding [51].

### 3.6. Thawing Loss

**Figure 5:**
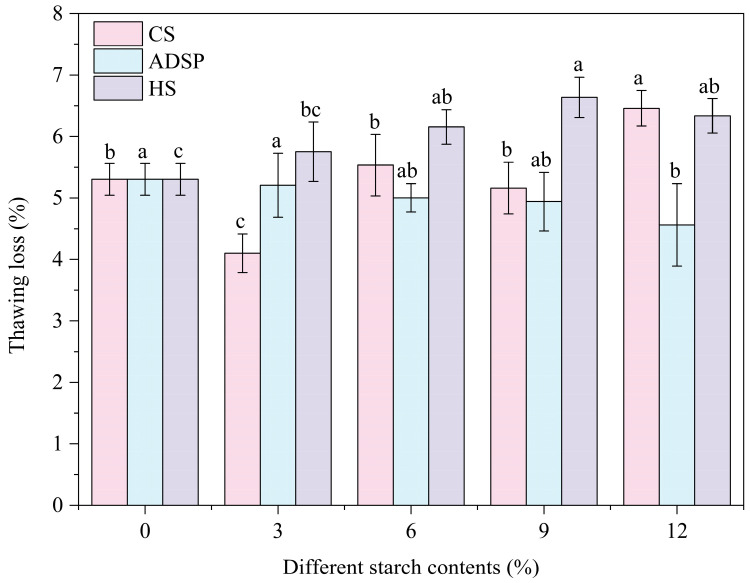
Effect of different starch contents on thawing loss of meat analogs. Note: Different lowercase letters indicate significant differences in thawing loss among different starch contents in the same starch (*p* < 0.05).

The water-holding capacity of the proteins was determined by measuring the thawing loss (Figure 5). The thawing loss was lowest (4.16%) with the content of 3% CS, and as the amount of ADSP increased, the thawing loss continuously decreased, whereas with further increases in CS and HS, the thawing loss increased. At low content levels, the 3% CS showed the best water-holding capacity. ADSP provided a pathway for a stable improvement in the water-holding capacity with the increasing concentration.

A low thawing loss rate indicates that the product can lock in water during freeze–thaw cycles. This mechanism is closely linked to the LF-NMR analysis, and the water-holding capacity of the system primarily relies on the interconnected porous structure generated through the interaction between starch and protein molecules. This network physically traps a large number of water molecules, such as immobile water (high M_21_), through capillary forces and steric hindrance, thereby preventing their loss. The hydrophilic groups on starch and protein molecular chains (such as hydroxyl and phosphate groups) can form hydrogen bonds with water molecules, chemically fixing water as bound water (high M_2b_). With its hydrophilic phosphate groups and dense network, ADSP comprehensively reduces water mobility; thus, its water-holding capacity improves with increasing concentrations. CS (3%) exhibited a good performance, possibly because it significantly increased the proportion of the hardest-to-lose bound water (M_2b_ was highest) without the excessive interference of the protein network. The increased thawing loss with high levels of CS and HS is a macroscopic manifestation of the supersaturation effect: an excessive amount of starch competes with protein for water and may disrupt the integrity of the protein network due to its high degree of swelling, leading to a decrease in the overall water-holding capacity. Therefore, adding a small amount of starch helps to reduce the freeze–thaw loss of meat analogs and avoid the decline of product quality during storage, transportation, and subsequent processing.

### 3.7. Emulsifying Properties

**Figure 6:**
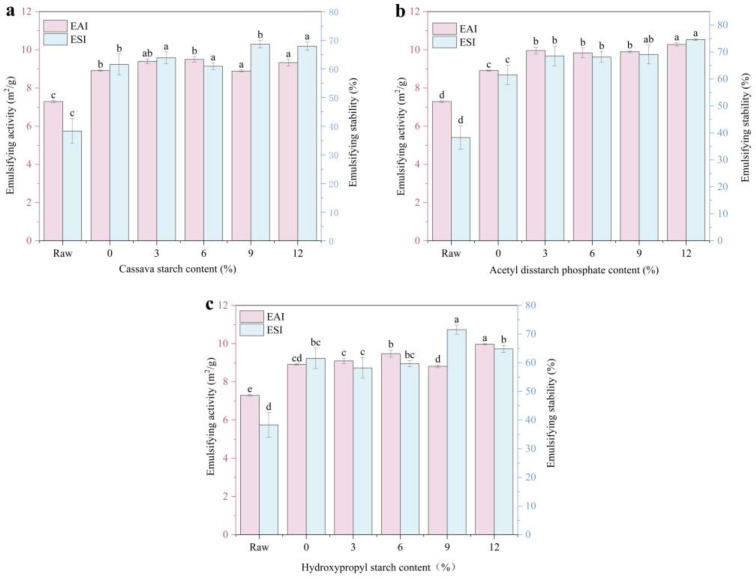
Effect of different starch contents on emulsifying characteristics of meat. (**a**) CS; (**b**) ADSP; and (**c**) HS. Note: Different lowercase letters indicate significant differences in emulsifying characteristics among different starch contents in the same starch (*p* < 0.05). EAI, emulsifying activity index; ESI, emulsifying stability index.

The adsorption capacity of the proteins at the interface was determined by measuring their emulsification properties (Figure 6). Compared to the raw material, extruded meat analogs exhibited a significantly higher emulsifying activity (increasing from 7.28 to 8.91 m^2^/g) and stability (from 38% to 62%). The emulsifying activities of histones with 3% and 6% starch added did not change significantly. The emulsifying activity of histones with 9% CS and HS added decreased slightly, but their emulsifying stability increased significantly. With an increase in the ADSP content, the emulsifying activity and stability of the meat analogs increased. The emulsifying performance of the 12% ADSP was the highest at 10.28 m^2^/g and 75%, respectively.

Tang and Sun [52] observed that surface hydrophobia influences protein emulsification, where globular protein unfolding exposes hydrophobic residues and alters the interfacial protein rearrangement. Starch undergoes gelatinization and degradation during HME processing, producing reducing sugars (such as dextrins and oligosaccharides) that react with the amino groups of proteins in a Maillard reaction [39]. This may expose more hydrophobic regions of the protein, and this structural change enhances the affinity between the protein and oil droplets, thereby promoting better emulsification [53]. The more starch that is added, the more enhanced the mutual aggregation of the exposed hydrophobic groups, which affects the binding and adsorption of proteins at the oil–water interface; thus, the emulsification stability is gradually reduced. Consequently, the starch content greatly affects the emulsifying properties of meat analogs.

ADSP performed the best, and the reasons for this may be multifaceted. First, it induces the strongest protein conformational changes (as shown by UV spectroscopy), possibly by exposing effective hydrophobic regions. Second, the ordered, highly cross-linked protein network formed (as shown by DSC and SDS-PAGE) could form a mechanically strong interfacial film on the oil droplet surface [54]. Finally, the hydrophilicity of its phosphate groups may help the entire protein–starch complex to remain well dispersed in the aqueous phase, thus effectively stabilizing the emulsion, which is conducive to the application of products in food processing.

### 3.8. IVPD

**Figure 7:**
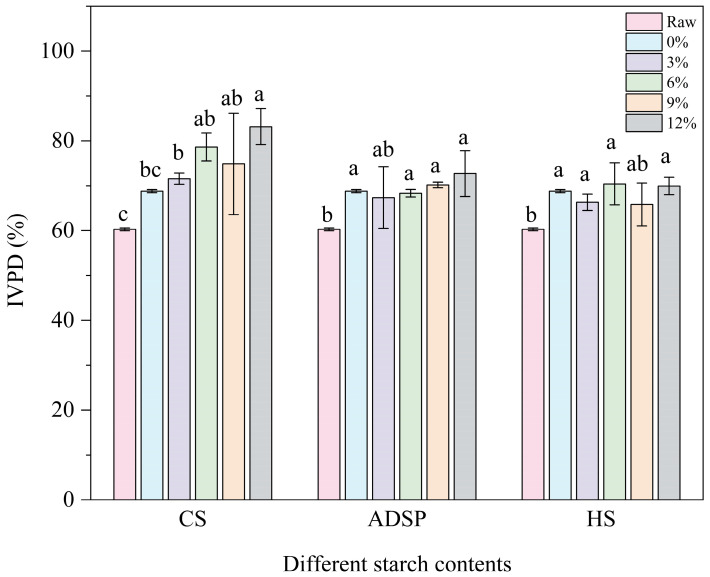
In vitro protein digestibility (IVPD) of meat analogs with different starch contents. Note: Different lowercase letters indicate significant differences in IVPD among different starch contents in the same starch (*p* < 0.05).

The digestibility of proteins is shown in Figure 7. The extrusion process significantly increased the protein digestibility from 60% in the raw material to 69%. The ADSP and HS content had no further significant effects. However, the CS continuously and significantly increased the digestibility in a dose-dependent manner (12%) and increased the digestibility to an impressive 83%, making it the most significant additive for improving protein digestibility.

Protein digestibility depends on whether digestive enzymes (such as pepsin and trypsin) can effectively access and hydrolyze peptide bonds [55]. HME enhances digestibility in two ways: destroying the complex tertiary and quaternary structures of proteins through thermal denaturation, exposing the peptide bonds that were originally folded inside, and increasing the accessibility of enzyme cleavage sites [56]. Moreover, high temperatures can inactivate naturally occurring anti-nutritional factors in plant materials, such as protease inhibitors, thus removing the inhibition of digestive enzymes [48]. CS can significantly improve digestibility, primarily through its high amylopectin content. The literature indicates that amylopectin is more prone than amylose to degradation into short-chain dextrins and oligosaccharides during extrusion. This study speculated that these degradation products may interact with proteins (e.g., through the Maillard reaction) or, through physical entanglement, prevent proteins from over-aggregating into dense, hard, and enzyme-impenetrable blocks during cooling. Instead, CS may help form a relatively loose, flexible network. This flexible structure makes protein chains more easily accessible to enzymes in a simulated digestive environment, thus greatly improving digestibility [57]. This elegantly explains a potential paradox: why can an additive that promotes protein aggregation (as shown by spectral analysis) improve digestibility? The key to the answer is not whether to aggregate but how to aggregate; CS guides proteins to form a functionally favorable (digestible) aggregated state.

## 4. Conclusions

By systematically analyzing the interactions between different starches and plant proteins during high-moisture extrusion, this study has revealed a multi-level, interconnected network of structure–function relationships. The starch content significantly affected the functional and structural properties of meat analogs. At 3% CS, the most bound water was released, thereby significantly reducing the thawing loss to 4.16%. Notably, the 3% CS significantly improved the in vitro digestion rate of proteins by increasing the protein content. At 12%, the CS achieved a digestion rate of 83%, which is crucial for enhancing the nutritional bioavailability of plant-based products. ADSP was the only starch that significantly reduced the T_2b_ (from 0.45 to 0.37 ms) and T_22_ (from 205.61 to 175.30 ms). Via the charged phosphate group, CS effectively promoted the formation of an orderly and tight cross-linking network of proteins, which not only significantly improved the water retention of the product but demonstrated good emulsification (12% ADSP provided the highest EAI 10.28 m^2^/g and ESI 75%), making it valuable for building a stable lotion system. Furthermore, the hydroxypropyl group of HS may stabilize the secondary structure of proteins via hydrogen bonding and hydrophobic interactions, thereby forming a high-viscosity protective matrix. The Tp increased from 123.58 to 125.71 °C, significantly improving the product’s ability to resist high temperatures. Although ADSP increased the ΔH, it sacrificed the Tp, whereas HS achieved a balance between thermal stability and structural orderliness. Our findings provide insights into the influence of modified starch on the structural and functional characteristics of meat analogs, thereby expanding their potential applications in meat analog production. Future research should investigate the influence of modified starch on the flavor characteristics of meat analogs and the effects of modified starches derived from other starch sources (e.g., corn starch and potato starch) on the properties of meat analogs.

## Figures and Tables

**Table 1:** Effects of different starch contents on T_2_ relaxation time of meat analogs.

Starch Contents (%)	Starch-Free	6% CS	6% ADSP	6% HS
T_2b_ (ms)	0.45 ± 0.01 ^a^	0.44 ± 0.02 ^ab^	0.37 ± 0.01 ^c^	0.43 ± 0.01 ^b^
T_21_ (ms)	15.71 ± 0.04 ^a^	14.71 ± 0.06 ^b^	13.69 ± 0.95 ^c^	15.6 ± 0.30 ^ab^
T_22_ (ms)	205.61 ± 3.73 ^b^	251.42 ± 0.93 ^a^	175.30 ± 3.71 ^c^	252.76 ± 2.49 ^a^
M_2b_ (%)	6.15 ± 0.08 ^b^	7.03 ± 0.06 ^a^	4.74 ± 0.10 ^d^	5.55 ± 0.11 ^c^
M_21_ (%)	91.87 ± 0.10 ^b^	91.38 ± 0.11 ^b^	93.16 ± 0.11 ^a^	93.04 ± 0.08 ^a^
M_22_ (%)	2.04 ± 0.07 ^a^	1.52 ± 0.08 ^b^	2.12 ± 0.09 ^a^	1.46 ± 0.09 ^b^

Note: Comparisons were performed between values in the same row; values with different letters indicate a significant difference at *p* < 0.05.

**Table 2:** Effects of different starch contents on thermal transition temperature and enthalpy of meat analog products.

Different Starch Contents		Tp (°C)	ΔH (J/g)
CS (%)	0	123.58 ± 2.72 ^a^	28.69 ± 0.06 ^ab^
	3	123.05 ± 0.77 ^a^	29.54 ± 1.15 ^a^
	6	123.68 ± 2.53 ^a^	29.94 ± 1.29 ^a^
	9	123.55 ± 0.93 ^a^	27.65 ± 0.19 ^b^
	12	123.42 ± 1.27 ^a^	28.89 ± 0.01 ^ab^
ADSP (%)	0	123.58 ± 2.72 ^a^	28.69 ± 0.06 ^d^
	3	122.12 ± 0.62 ^a^	38.1 ± 0.25 ^a^
	6	122.07 ± 1.42 ^a^	34.49 ± 1.19 ^bc^
	9	123.69 ± 1.59 ^a^	35.18 ± 1.67 ^b^
	12	121.71 ± 0.36 ^a^	32.94 ± 0.8 ^c^
HS (%)	0	123.58 ± 2.72 ^b^	28.69 ± 0.06 ^a^
	3	123.53 ± 1.16 ^b^	28.52 ± 0.87 ^a^
	6	125.71 ± 1.61 ^a^	29.04 ± 2.16 ^a^
	9	122.70 ± 1.05 ^b^	27.25 ± 1.02 ^ab^
	12	124.57 ± 0.20 ^ab^	27.56 ± 0.33 ^ab^

Note: Comparisons were performed between values among different starch contents in the same starch; values with different letters indicate significant differences at *p* < 0.05.

## Data Availability

Data will be made available on request.

## Abbreviations

The following abbreviations are used in this manuscript:

ADSP	acetyl distarch phosphate
CS	cassava starch
EAI	emulsifying activity index
ESI	emulsifying stability index
HME	high-moisture extrusion
HS	hydroxypropyl starch
IVPD	in vitro protein digestibility
LF-NMR	low-field nuclear magnetic resonance
SDS-PAGE	sodium dodecyl sulfate–polyacrylamide gel electrophoresis
Tp	peak temperature
UV	ultraviolet
